# Expanding the Reach of Research: Quantitative Evaluation of a Web-Based Approach for Remote Recruitment of People Who Hear Voices

**DOI:** 10.2196/23118

**Published:** 2021-06-03

**Authors:** Benjamin Buck, Ayesha Chander, Rachel M Brian, Weichen Wang, Andrew T Campbell, Dror Ben-Zeev

**Affiliations:** 1 Behavioral Research in Technology and Engineering (BRiTE) Center Department of Psychiatry and Behavioral Sciences University of Washington Seattle, WA United States; 2 Department of Computer Science Dartmouth College Hanover, NH United States

**Keywords:** digital health, research procedures, recruitment, mobile phone

## Abstract

**Background:**

Similar to other populations with highly stigmatized medical or psychiatric conditions, people who hear voices (ie, experience auditory verbal hallucinations [AVH]) are often difficult to identify and reach for research. Technology-assisted remote research strategies reduce barriers to research recruitment; however, few studies have reported on the efficiency and effectiveness of these approaches.

**Objective:**

This study introduces and evaluates the efficacy of technology-assisted remote research designed for people who experience AVH.

**Methods:**

Our group developed an integrated, automated and human complementary web-based recruitment and enrollment apparatus that incorporated Google Ads, web-based screening, identification verification, hybrid automation, and interaction with live staff. We examined the efficacy of that apparatus by examining the number of web-based advertisement impressions (ie, number of times the web-based advertisement was viewed); clicks on that advertisement; engagement with web-based research materials; and the extent to which it succeeded in representing a broad sample of individuals with AVH, assessed through the self-reported AVH symptom severity and demographic representativeness (relative to the US population) of the sample recruited.

**Results:**

Over an 18-month period, our Google Ads advertisement was viewed 872,496 times and clicked on 11,183 times. A total amount of US $4429.25 was spent on Google Ads, resulting in 772 individuals who experience AVH providing consent to participate in an entirely remote research study (US $0.40 per click on the advertisement and US $5.73 per consented participant) after verifying their phone number, passing a competency screening questionnaire, and providing consent. These participants reported high levels of AVH frequency (666/756, 88.1% daily or more), distress (689/755, 91.3%), and functional interference (697/755, 92.4%). They also represented a broad sample of diversity that mirrored the US population demographics. Approximately one-third (264/756, 34.9%) of the participants had never received treatment for their AVH and, therefore, were unlikely to be identified via traditional clinic-based research recruitment strategies.

**Conclusions:**

Web-based procedures allow for time saving, cost-efficient, and representative recruitment of individuals with AVH and can serve as a model for future studies focusing on hard-to-reach populations.

## Introduction

### Background

Auditory verbal hallucinations (AVH; or *voices*), or erroneous perceptions of speech, are a hallmark symptom of schizophrenia spectrum disorders (SSDs). They are experienced by 5% to 28% of the general population [1-4], including many people with a range of psychiatric diagnoses as well as many people who are otherwise considered healthy. In their most severe form, AVH are linked to anxiety, depression, and functional impairment [5,6]. AVH present unique challenges for research. First, individuals with SSDs who hear voices are considered a hard-to-reach population or a group that faces geographical, social, or economic barriers to participation [7,8]. SSDs are overrepresented in economically deprived and socially isolated groups [9,10], and treatment resources for them are scarce [11]. Many have no contact with mental health providers [12-14]. Others are reluctant to engage with the mental health system because of concerns about stigma [15], disagreement with diagnosis [16,17], or negative attitudes toward services [18]. Second, common clinical research practices often fail to access individuals with less severe or non-clinical AVH. Most individuals with AVH do not meet the criteria for an SSD; they often present with a range of diagnoses, including major depressive disorder, bipolar disorder, or may have never received a diagnosis at all [19,20]. Due to their reduced impact and increased independent coping skills, individuals with nonclinical AVH may be less likely to engage in research through traditional clinical settings. They may also opt against self-identifying because of concerns about stigma [21].

Digital technologies may help address these barriers. The average American adult is estimated to spend 3 hours and 48 minutes per day on a smartphone, tablet, or computer, with the majority of that time (62%) spent on the internet and using an app on a smartphone [22]. More than 1 in 3 adults report that they have gone on the internet to better understand a health condition [23]. Although smartphone ownership tends to lag behind the general population, many individuals with serious mental illnesses report owning a smartphone [24], and this number has rapidly increased over the past two decades [25]. Individuals with SSDs report similar levels of health-related internet and web-based engagement [26] and are willing to engage in a variety of research or clinical activities on the web, including assessments [27,28], interventions [29-31], and peer communication [32,33]. Web-based approaches allow participants to covertly self-identify and thus could reduce the impact of stigma on research participation [34]. Furthermore, the COVID-19 pandemic has revealed additional vulnerabilities in in-person research recruitment procedures. During the pandemic, many of these activities have halted; however, research engagement through digital technology can continue while remaining adherent to physical distancing guidelines.

One particularly promising approach to address these issues is the use of technology-assisted remote research. This term describes a constellation of web-based tools (eg, advertisements posted in search engine results and email listservs) that require no face-to-face interactions with prospective participants and thus obviate many of the barriers common in traditional research. These differ from traditional recruitment methodologies that require partnerships with partner organizations with brick-and-mortar locations or other recruitment tools such as flyers. These efficient and cost-effective [35,36] tools have been used to recruit research participants from several psychiatric populations, including people with depression [35], bipolar disorder [36], and suicidality [37]. These tools may be specifically well suited to address the extant challenges in recruiting and engaging individuals who experience AVH. As it is not situated within a health care system, technology-assisted remote research may be less susceptible to overrepresenting individuals engaged in treatment. It also potentially removes economic and social barriers faced by hard-to-reach populations (eg, time constraints and travel time) and allows for anonymous participation, thus reducing the impact of stigma.

Web-based methodologies are not without challenges. Research has examined the extent to which web-based research participants (eg, users of web-based platforms such as Amazon Mechanical Turk) are representative of the general population [38-40]. Others have raised concerns about participants’ *gaming* or earning payments without honest and effortful participation [41]. For example, participants may attempt to complete web-based studies multiple times by masking their identifiers (eg, name and email address). Few studies have presented methods and models to engage difficult-to-reach populations while preserving the security, privacy, and validity of research data.

### Objectives

In this paper, we report on the use of a technology-assisted remote research approach designed to recruit people who experience AVH. These procedures combine public-facing technologies (eg, Google Ads) with automated digital tools (eg, coding scripts to filter prospective participants) and available remote human support to efficiently recruit a difficult-to-reach population while preserving data security and quality. We describe the structure of that system and report several metrics assessing its performance, including (1) the number of web-based advertisement impressions (ie, number of times the web-based advertisement was viewed); (2) clicks on that advertisement; (3) engagement with web-based research materials; and (4) the extent to which it succeeded at representing a broad sample of individuals with AVH, assessed through self-reported AVH symptom severity and demographic representativeness (relative to the US population) of the sample recruited.

## Methods

### Overview

These digital tools were built for a primary study described elsewhere examining the real-time, real-place phenomenology of AVH [42]. This involved downloading and carrying a smartphone app for a 30-day study period that deployed brief ecological momentary assessment questionnaires and captured data through smartphone sensors. Study inclusion criteria included speaking English, being 18 years or older, living in the United States, experiencing AVH at least once per week, and ownership of an Android smartphone. Exclusion criteria included previous participation in the study and being unavailable for 30 days of consecutive data collection. The internal review boards at the University of Washington and Dartmouth College approved all the study procedures.

### Google Ads and Keywords

To recruit individuals reporting AVH, we used Google Ads, a system of web-based advertisements wherein advertisements are viewable by individuals who use terms that match preselected keywords provided by the advertiser. Advertisements are designed to resemble a typical Google search result and thus comprise a title, URL, and brief description. In addition to keywords, the system allows for the entry of *negative keywords,* which function in the opposite way, ensuring that the advertisements are not viewable by individuals who search one of the negative keywords. Google Ads records the number of impressions, keywords used, number of clicks linked to the landing page and cost per click, and basic demographics of those who clicked on the advertisement (eg, age, gender, and household income, if reported to Google by the user). For this study, we selected keywords based on several sources: qualitative responses during our early work [43] as well as a rapid review of the academic literature, consultation with researchers focused on serious mental illness, and a review of blogs of people with lived experience. These keywords included descriptors that involved both clinical language (eg, *schizophrenia* and *bipolar disorder*) and nontraditional appraisals of AVH or related terms (eg, *talking to ghosts* and *going crazy*). Positive keywords were allowed to have a *broad match* with search terms, according to which search terms could be reordered or accompanied with other terms. Negative keywords were entered with a *phrase match* setting, according to which the exact phrase must be included in the search for the user to be blocked. Negative keywords were selected to prevent individuals from attempting to complete the study a second time (eg, *hearing voice research* and *bipolar studies that pay)*.

### Web-Based Recruitment Materials

**Figure 1:**
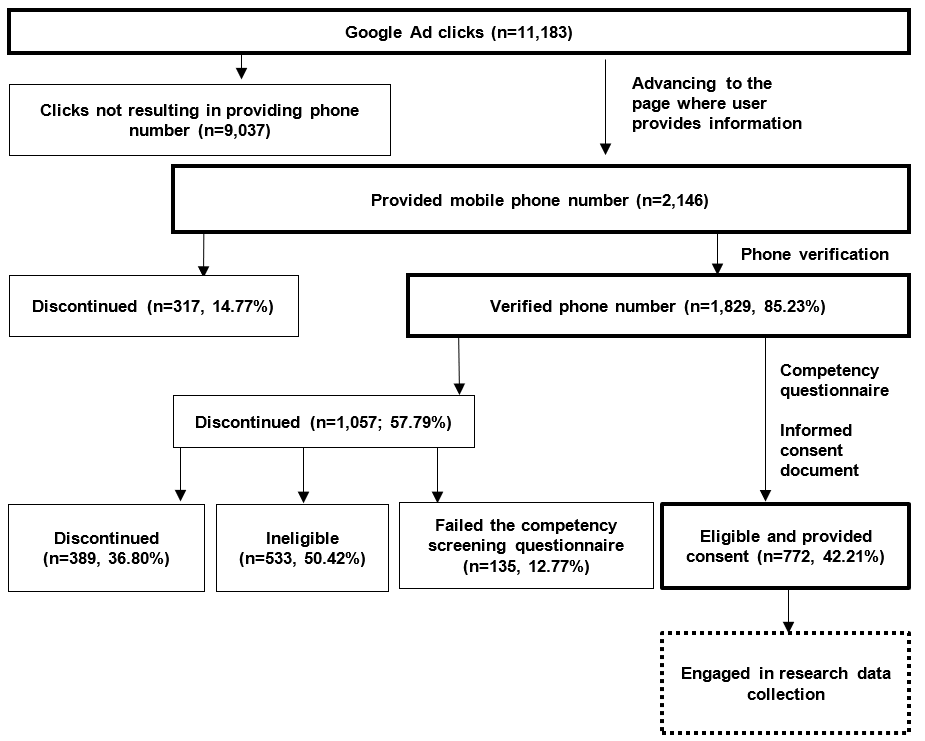
Participant flow and attrition at each stage of the web-based engagement procedure.

Once users clicked on the advertisement, they were taken to the study landing page that provided written and video descriptions of the study. The consent form was available for download on this page. Participants were instructed to click on a “see if I am eligible” button if they were interested; this button then triggered a pop-up message requesting their email and a valid mobile phone number. This number received a text message with a code that prospective participants had to enter on the study website to gain access to the eligibility questionnaire. Once found eligible, participants were again given access to the consent form to download and review and asked to answer all competency questions correctly within 3 attempts. Participants were excluded if they were unable to complete this step successfully, as they were deemed unable to provide informed consent. Participants were also excluded if they had previously participated in the study. Therefore, the server ran the email and phone numbers provided through the participant database and automatically blocked people with repeat phone numbers from continuing the enrollment process. If participants met eligibility criteria via the screening questions and provided informed consent, their participation in the study officially began (ie, access to baseline assessments and the study app was provided). If at any point their responses indicated that they were ineligible (eg, 3 failed attempts to answer competency questions or previous participation), they were taken to a page where they were thanked for their interest but informed that they were ineligible to participate. Throughout this process, a staff member was available by phone and email for participants to call if the prospective participant faced barriers or had questions. Figure 1 provides an overview of the flow of interested individuals from the landing page to the consent and initiation of the study.

### Data Security and Validity Procedures

In addition to the phone verification process, other procedures were also used to deter *gamers* or participants aiming to earn payments through duplicate or otherwise dishonest participation. These involved identifying prospective participants based on suspicious digital activity; when such users were identified, a research coordinator contacted them to conduct additional live screening by telephone. We identified this suspicious activity by (1) reviewing their means of access to the study landing page and (2) assessing duplicate emails, phone numbers, and email addresses. First, as a user cannot ensure that a Google Ad will appear in their search terms, it was expected that gamers would attempt to access the study landing page by entering the study URL directly, as opposed to honest participants who would only respond to the link listed in Google Ads. Thus, different links distinguished those who accessed the page via Google Ads versus those who accessed it directly. When a direct arrival initiated the screening process, a research coordinator would receive a notification and conduct additional screening of the prospective participant by phone; they were only allowed to proceed if cleared by the research coordinator. Second, the research coordinator would also receive an automated email if a participant attempted to enter the study with (1) an identical IP (internet protocol) address to a previous participant or (2) an email address for which characters matched the email of a previously enrolled participant by >90%. The study responses of these suspicious participants were reviewed on an ongoing basis (ie, identifiers and patterns in survey responses) and discussed at subsequent team meetings to determine whether additional screening was warranted by these patterns of responses. Participants were discontinued from the study if it was determined after additional screening that they were attempting to participate in the study for a second time (n=44) or were otherwise dishonest about their eligibility (n=2). No adverse events occurred throughout the course of the study.

## Results

### Overview

The aim of this study is to examine the efficiency and effectiveness of web-based recruitment of individuals who experience AVH, according to a number of metrics, including (1) the number of web-based advertisement impressions (ie, number of times it was viewed) and clicks; (2) engagement with web-based research materials (ie, whether and how participants enrolled); and (3) the extent to which it succeeded in representing a broad sample of individuals with AVH, assessed through self-reported AVH symptom severity and demographic representativeness (relative to the US population).

### Advertisement Impressions and Clicks

Advertisements were posted from February 2018 to September 2019, with a total budget of US $4429.25. The advertisement was viewed 872,496 times and was clicked on 11,183 times, reaching a click-through rate (clicks per impression) of 1.28% at an average cost of US $0.40 per click. The cost of a successfully recruited participant, that is, one who completed the screening processes and informed consent portions completely, was US $5.74 per person. The top 5 advertisement keywords were *mental illness*, *schizophrenia*, *mental health*, *bipolar*, and *hear voices*. Table 1 presents the number of impressions, clicks, click-through rate, and cost per click by advertisement keyword. Of the individuals who clicked on the advertisement and had available demographic information, most (9896/11,223, 88.18%) clicked on the advertisement through mobile phones, whereas few used desktop computers (700/11,223, 6.24%) or tablets (627/11,223, 5.59%).

**Table 1:** Measures of advertisement engagement for all keywords included to reach people who hear voices^a^.

Keywords	Impressions^b^, n	Clicks, n	Click-through rate (%)	Average cost per click (US $)	Total cost (US $)
Mental illness	188,766	2263	1.20	0.42	951.33
Schizophrenia	97,872	1945	1.99	0.41	794.19
Mental health	146,438	1753	1.20	0.43	757.05
Bipolar	82,815	931	1.12	0.44	410.64
Hear voices	59,321	881	1.49	0.33	294.16
I hear voices	55,629	815	1.47	0.35	286.64
Hearing sounds	47,702	610	1.28	0.37	226.86
Hearing voices in head	22,234	445	2.00	0.34	151.06
Mental illness hearing voices	19,685	422	2.14	0.35	148.30
Schizoaffective	8787	181	2.06	0.39	70.28
Hearing voices disorder	9972	175	1.75	0.39	67.77
Auditory hallucinations	6479	158	2.44	0.33	51.59
Hearing things	39,836	148	0.37	0.33	48.90
Delusional	22,321	130	0.58	0.33	42.88
Going crazy	11,880	78	0.66	0.33	25.49
Talking to ghosts	9904	67	0.68	0.37	24.96
Stress relief	21,329	64	0.30	0.46	29.12
Spirits talking	13,142	58	0.44	0.42	24.09
Musical ear syndrome	3767	19	0.50	0.44	8.39
Am I crazy	1644	19	1.16	0.31	5.95
Am I insane	942	12	1.27	0.45	5.43
Disembodied voices	227	3	1.32	0.28	0.84
I’m not crazy	1591	3	0.19	0.39	1.18
Hearing voices^c^	122	3	2.46	0.71	2.15
Voice hearing^c^	91	0	0	0	0

^a^The total number of impressions was 872,496; the total number of clicks was 11,183; average click-through rate was 1.28%; average cost per click was US $0.40; and the total cost was US $4429.25.

^b^An impression is counted each time the advertisement is shown on a search result page.

^c^These keywords were included for only a portion of the study period because of concerns related to repeat study participants searching for and finding the study advertisement.

### Study Enrollment

The flow of interested users from Google Ads through informed consent is shown in Figure 1. Of the 11,183 clicks on the study Google Ad, a total of 2146 individuals provided their mobile phone number to be verified for participation in the study. Of the 2146 participants who provided a phone number, 1829 (85.23%) verified this phone number with a code. Of the 1829 individuals with a verified phone number, 772 (42.21%) provided consent to participate in the study. Most individuals who verified their phone number but did not provide informed consent, did not meet the eligibility criteria (533/1057, 50.43%), whereas the remainder either failed a competency screening questionnaire (135/1057, 12.77%) or chose not to participate (389/1057, 36.8%).

### Participant AVH Frequency and Severity

Results related to participants’ AVH frequency and severity are presented in Table 2. Most individuals who provided consent to participate reported that they had sought treatment for their AVH at some point in their lifetime (492/756, 65.1%) but over one-third had not (264/756, 34.9%). The most common self-reported diagnoses were depression (488/749, 65.2%), bipolar disorder (352/749, 47%), and posttraumatic stress disorder (328/749, 43.8%). Most participants reported hearing voices at least once a day (666/756, 88.1%) and that the voices interfered with their daily activities in some way (697/755, 92.4%), with almost half of the participants reporting “quite a bit” or “extreme” interference (346/755, 45.9%). Most reported that the voices they hear are distressing (689/755, 91.3%) and more than half reported “quite a bit” or “extreme” distress (416/755, 55.1%). Participants varied in terms of how open they were about these voices with others. Most participants (443/756, 58.6%) had shared their experience of voices with a medical professional. Approximately half of the participants had immediate family members (376/755, 49.8%) or a significant other (350/754, 46.4%) who knew about their experience of voices. One-tenth (75/753, 10%) of the sample reported that no one else knew about their experience of voices. These figures suggest that this remote recruitment strategy successfully engaged a broad continuum of people with AVH experiences, including individuals with significant and severe AVH as well as those who had never engaged in treatment for them.

**Table 2:** Participants’ diagnoses, experience, frequency, severity, and behavioral interference of auditory verbal hallucinations.

Clinical characteristics		Participants, n (%)
**Lifetime treatment seeking (n=756)**		
	Yes	492 (65.1)
	No	264 (34.9)
**Self-reported diagnosis^a^**		
	Alzheimer or Parkinson disease (n=747)	4 (0.5)
	Bipolar disorder (n=749)	352 (47.0)
	Depression (n=749)	488 (65.2)
	Traumatic brain injury (n=746)	50 (6.7)
	Migraines (n=747)	141 (18.9)
	Schizoaffective disorder (n=748)	210 (28.1)
	Schizophrenia (n=749)	208 (27.8)
	Posttraumatic stress disorder (n=749)	328 (43.8)
	Substance use (n=747)	228 (30.5)
	Seizures (n=748)	54 (7.2)
	None of the above (n=749)	61 (8.1)
	Other (n=772)	87 (11.3)
**Who knows about your voices?^a^**		
	No one knows (n=753)	75 (10.0)
	People you know online but not in person (n=756)	63 (8.3)
	Medical professionals/primary doctor/therapist (n=756)	443 (58.6)
	Significant other (ie, boyfriend, girlfriend, or spouse) (n=754)	350 (46.4)
	Some of my friends (n=756)	333 (44.0)
	All my friends (n=755)	83 (11.0)
	Extended family (n=756)	105 (13.9)
	Immediate family (n=755)	376 (49.8)
**How frequently do you hear a voice or voices? (n=756)**		
	No voices	2 (0.3)
	Less than once day	88 (11.6)
	Once or twice a day	199 (26.3)
	Several times a day	242 (32.0)
	All the time/constantly	225 (29.8)
**How much do the voices interfere with your daily activities? (n=755)**		
	No interference	58 (7.7)
	A little bit	162 (21.5)
	Moderately	189 (25.0)
	Quite a bit	187 (24.8)
	Extremely interfering	159 (21.1)
**How distressing are the voices that you hear? (n=755)**		
	No voices are distressing me	66 (8.7)
	A little bit	116 (15.4)
	Moderately	157 (20.8)
	Quite a bit	205 (27.2)
	Extremely distressing	211 (27.9)

^a^Participants could select multiple options.

### Participant Demographics and Representativeness

The demographics of the study sample (Table 3) broadly reflect a number of US population trends. The average age of consented participants was 38.14 years (SD 9.86), which closely mirrored the median US age of 38.2 years [44]. In comparison with population estimates, the sample of individuals providing consent included larger percentages of multiple populations that are traditionally underrepresented in research, including Black or African American participants (108/760, 14.2% in our study, estimated at 12.7% of the US population [45]), multiracial participants (90/760, 11.8% vs 3.4% of the US population [45]), sexual minorities (156/766, 20.3% gay, lesbian, or bisexual vs 5% [46]; 12/771, 1.6% transgender vs 0.6% [47]), and the homeless (69/767, 9.0% vs 0.2% [48]). Other groups appeared underrepresented relative to the general population, including individuals who identified as Asian (9/760, 1.2% vs 5.6% [45]), Hispanic or Latino (101/765, 13.2% vs 18.3% [45]), and male (292/771, 37.9% vs 49.2% [45]).

**Table 3:** Demographics of participants who provided consent to participate.

Demographic characteristic			Values
Age (years; n=768), mean (SD)			38.14 (9.86)
**Sex or gender (n=771), n (%)**			
	Female		470 (61.0)
	Male		286 (37.1)
	**Transgender**		12 (1.6)
		Transgender woman	6 (0.8)
		Transgender man	6 (0.8)
	Other		3 (0.4)
**Race (n=760), n (%)**			
	White		537 (70.7)
	Black or African American		108 (14.2)
	Pacific Islander		2 (0.3)
	American Indian or Alaskan Native		14 (1.8)
	Asian		9 (1.2)
	More than one race		90 (11.8)
**Hispanic (n=765), n (%)**			
	Yes		101 (13.2)
	No		664 (86.8)
**Sexual orientation (n=766), n (%)**			
	Heterosexual or straight		591 (77.2)
	Gay or lesbian		50 (6.5)
	Bisexual		106 (13.8)
	Other		19 (2.5)
**Housing status (n=767), n (%)**			
	Independent or living on my own		348 (45.4)
	Living with family		313 (40.8)
	Assisted or supported living		33 (4.3)
	Substance treatment institution		4 (0.5)
	Homeless		69 (9.0)

## Discussion

### Principal Findings

Digital technologies are rapidly reshaping mental health research and services. One particular benefit of these approaches is their capacity to increase the reach of research to underserved populations. Previous work has demonstrated the potential of web-based engagement to facilitate help seeking among individuals at risk for psychosis by linking them to web-based resources about symptoms and treatments (eg, the *Strong365* campaign [49]). This study builds on this earlier work by demonstrating that web-based tools can remotely facilitate research participation of individuals with psychotic symptoms at both clinical and nonclinical levels. The recruited sample reported clinically significant experiences with voices that were frequent, distressing, and functionally interfering, and over one-third had never received treatment for them. Overall, the results suggest that these web-based procedures allow for the efficient, affordable, and representative recruitment of research participants without reliance on a clinical setting.

Web-based digital recruitment methods compare favorably with extant approaches for engaging hard-to-reach populations in several ways. First, such methods appear to reduce costs. Traditional recruitment methods incur several costs in the process of raising interest and awareness of a study, such as increased staff time, flyers, mailings, and presentations to clinical or educational facilities [50]. These needs are obviated through remote recruitment. Our web-based advertisements were viewed by over 870,000 individuals for 18 months for less than US $4500, a fraction of the cost that such exposure would necessitate using offline approaches. The approach of this study appeared to reduce costs relative to previous work using social media to recruit individuals with psychotic experiences [43]. Second, beyond increasing efficiency, these methods may improve representative sampling when the members of a group face barriers to participation. Research in other health-related populations has demonstrated that digital technologies may address these barriers [7,8,51], in particular, accessing broader geographic regions [52,53] and more diverse respondents [35,54]. This study provides support for these benefits and suggests that they may also reduce barriers specific to individuals on either end of the continuum of AVH severity. Individuals with psychosis are difficult to engage in clinical research, given social isolation and economic hardship [10,12,13]. Those with undiagnosed and untreated AVH may be even more difficult to engage, given a lack of contact with typical clinical research settings as well as potential concerns about self-identifying with stigmatized experiences. This approach appeared to engage participants from both ends of this continuum; one-third of the participants reported never seeking treatment for their voices, a similar proportion reported that they experienced highly distressing voices daily. This finding also provides additional support for the utility of these approaches to engage individuals at risk either in programs that encourage help seeking [49,55] or in remotely delivered interventions [33]. Although a few examples provide support for the feasibility of these initiatives, future work should examine remote treatment-seeking support and intervention for the psychosis continuum at a population level. Furthermore, notable in these data were the number of participants who were also members of other underrepresented populations, including racial minorities, sexual minorities, and the homeless. Although it remains an open question why these groups are better represented using these methods, it is possible that web-based engagement reduces barriers for underrepresented groups that may face additional intersectional experiences of stigma on top of those related to their mental health concerns alone.

### Limitations

This study had some limitations. Although web-based methods may ameliorate some recruitment biases, they may worsen others. Web-based recruitment strategies rely on participants’ ownership and adeptness to use technology to engage. Individuals who lack either may struggle to engage in these new avenues. This may be particularly pronounced among individuals who face economic barriers to several needed services. Most visitors to the research study landing page did not provide informed consent to participate. Specific factors may predict a lack of willingness to persist through automated screening steps (eg, persecutory ideation and cognitive functioning). If this is the case, this could limit the representativeness of the sample. Although our approach successfully engaged members of several typically underrepresented groups, others accounted for smaller proportions than their proportion of the US population, including Hispanic and Asian individuals. The prevalence of AVH in specific racial or ethnic groups is not clear in the academic literature at present; thus, it is not clear from these data whether overrepresentation of these groups relative to population demographic estimates is the result of a greater prevalence of AVH in specific groups, characteristics of our recruitment strategies, or other causes. Overall, however, our results suggest that web-based recruitment methods engage a diverse sample of individuals with AVH experiences.

### Conclusions

Technology-assisted remote research procedures address several barriers that are common in traditional research. Engaging prospective participants outside of typical clinical settings removes existing sampling biases, allowing for greater representation of psychiatric populations. These procedures have also been able to continue despite the halted in-person research activities in light of social distancing measures imposed by the COVID-19 pandemic. This expansion of access and reach has been a principal contribution of digital health technologies, and health care institutions have only just begun to witness their potential impact [56]. Just as digital technologies create considerable opportunities for treatment engagement, such tools can also enhance clinical research.

## Abbreviations

AVH: auditory verbal hallucinations
IP: internet protocol
SSD: schizophrenia spectrum disorder
